# Toward Precise Nutrient Value of Feed in Growing Pigs: Effect of Meal Size, Frequency and Dietary Fibre on Nutrient Utilisation

**DOI:** 10.3390/ani11092598

**Published:** 2021-09-04

**Authors:** Élisabeth Chassé, Frédéric Guay, Knud Erik Bach Knudsen, Ruurd T. Zijlstra, Marie-Pierre Létourneau-Montminy

**Affiliations:** 1 Department of Animal Science, Université Laval, 2425 Rue de l’Agriculture, Québec, QC G1V 0A6, Canada; Frederic.Guay@fsaa.ulaval.ca (F.G.); marie-pierre.letourneau-montminy.1@ulaval.ca (M.-P.L.-M.); 2 Department of Animal Science, Aarhus University, 8830 Tjele, Denmark; knuderik.bachknudsen@anis.au.dk; 3 Department of Agricultural, Food and Nutritional Science, University of Alberta, Edmonton, AB T6G 2P5, Canada; zijlstra@ualberta.ca

**Keywords:** meal size, meal frequency, feed intake, dietary fibre, digestibility, transit time, exogenous enzymes, growing pigs

## Abstract

**Simple Summary:**

Feed costs are the most important in swine production. Precise determination of nutritional values of pig diets can help reducing feed costs by reducing security margins for nutrients and therefore provide a more sustainable swine production. In commercial farms, pigs have free access to feed and eat with no limitation according to their natural behaviour. In contrast, during digestibility trials, pigs are restricted in their daily intake of feed, which is distributed in a limited number of meals. The number of meals per day and the amount of feed consumed daily can affect the digestibility of the nutrients, the transit time and the metabolism. To reduce feed costs, by-products are frequently added to diets. Most by-products are rich in dietary fibre, which are known to have negative effects on digestibility. Enzymes can be supplemented in the diet to counteract the negative aspects of dietary fibre, but their efficiency can vary depending on the number of meals per day and the amount of feed consumed daily.

**Abstract:**

Nutritional values of ingredients have been and still are the subject of many studies to reduce security margins of nutrients when formulating diets to reduce feed cost. In most studies, pigs are fed a limited amount of feed in a limited number of meals that do not represent how pigs are fed in commercial farm conditions. With free access to feed, pigs follow their intrinsic feeding behaviour. Feed intake is regulated by satiety and satiation signals. Reducing the feed intake level or feeding frequency can affect digestibility and transit time and induce metabolic changes. To reduce feed costs, alternative ingredients that are frequently rich in dietary fibre are added to diets. Fibre acts on the digestion process and transit time by decreasing energy density and causing viscosity. Various analyses of fibre can be realised, and the measured fibre fraction can vary. Exogenous enzymes can be added to counteract the effect of fibre, but digestive tract conditions, influenced by meal size and frequency, can affect the efficiency of supplemented enzymes. In conclusion, the frequency and size of the meals can affect the digestibility of nutrients by modulating gastrointestinal tract conditions (pH and transit time), metabolites (glucose and short-chain fatty acids) and hormones (glucagon-like peptide 1 and peptide tyrosine tyrosine).

## 1. Background

Feed cost represents 64 to 72% of the variable production cost in pig production [1]. Pork producers must also face volatile corn and soybean meal prices, the two main ingredients of diets in the USA and eastern Canada, concerning supply and demand. As a result, feed costs are expected to increase by 12% in 2021 [2]. China’s shortage of animal protein supply because of African swine fever has moved it to the position of the largest global importer of beef and pork. However, China’s animal protein imports are expected to decline in 2021, while its animal feed imports could surpass all-time highs in the 2020–2021 crop year. An increase in feed imports results in higher feed costs of animal protein sectors in North America, which face lower margins due to the COVID-19 crisis. This is one example, but many others could be provided and will occur in the future with periods of social crisis, health and climatic hazards.

Pig producers worldwide, therefore, seek low-cost alternatives, such as cereal by-products from the biofuel and milling industries, to feed their pigs to reduce feed costs [3]. Corn distillers dried grains with solubles (cDDGS), wheat middlings and bakery meal are by-products commonly used in pig diets [4,5]. Most of these by-products have a high energy and nutrient content but are fibrous [4]. The addition of by-products increases the intrinsic variation of the nutritional value of feedstuffs and may induce variation in animal response. The inclusion of by-products also induces significant changes in the feeding and requires optimising nutrient utilisation by pigs for sustainable swine production. With aiming to optimise nutrient utilisation, robust and flexible feed formulation needs to be developed while fine-tuning these three steps: (1) estimation of the nutritional value of ingredients considering modulating factors, (2) precise estimation of nutritional requirements and (3) precise systems to distribute the feed.

The nutritional value of feedstuffs can be predicted from its chemical composition that can be rapidly obtained with near-infrared spectroscopy (NIRS) or similar techniques. The relationships between proximal ingredient components (e.g., crude protein, neutral detergent fibres) and nutritive value were determined using digestibility trials (e.g., amino acids digestibility) followed by regression analyses [6] to complement those available in the literature [7]. It is assumed with these relationships that the nutritional value of feedstuffs is constant and unaffected by the animal, including feeding pattern.

The number and size of meals affect nutrient digestibility [8,9,10] and the dynamics of their metabolic availability. During digestibility trials, the daily feed allowance of pigs is usually restricted to a level below their maximum intake capacity. Generally, the daily feed allowance is provided to meet up to three times the metabolisable energy requirements for maintenance or 4% of the pig’s body weight [11]. This daily amount of feed is then distributed among one, two or three equal meals per day. However, pigs have ad libitum access to feed on commercial farms without limitations on intake or number of meals. Meal size and frequency can influence the digesta transit time, digestibility and thereby nutritional value of diets [8,9,12].

The gastrointestinal tract content is a chemically complex mixture of feed macromolecules, microorganisms and enzymes that interact to supply nutrients to the animal. Physicochemical characteristics, such as pH and transit time, can alter digestive processes, including the efficiency of digestive enzymes, nutrient absorption and microbial fermentation. These intestinal physicochemical characteristics are affected by many factors. Dietary fibre is of particular interest nowadays because of the increased dietary inclusion of fibre-rich alternative ingredients. For example, dietary fibre increases pancreatic secretion of bicarbonate in pigs [13]. Additionally, fibre, such as readily fermentable carbohydrates, can modify microbes throughout the gut and thus the production of lactic acid and short-chain fatty acids, which may, in turn, locally lower pH [14]. Fibre also acts on transit time [15,16]. However, the impact of fibre on the overall digestion process depends on its properties, especially its solubility, viscosity, fermentability, cross-linkages and lignification. With that of starch, the impact of dietary fibre is underappreciated in their complexity in pig nutrition [17].

Exogenous enzymes are frequently added to pig diets to increase the digestibility of phytic phosphorus (phytase) and fibre (e.g., xylanase). In addition to their specific action on phosphorus and fibre, these enzymes can positively affect overall nutritional value by enhancing the digestibility of minerals, amino acids and energy [18,19,20]. However, these effects remain variable, especially for xylanase. Part of this variation can be caused by the large dependency of enzyme activity and efficiency on the composition of the fibre matrix along with pH, transit time and other endogenous enzymes, such as proteases. These interactions are not fully understood and controlled.

Precision feeding systems develop quickly and represent a paradigm shift in pig feeding. To maximise the potential of precision feeding, all the steps of diet formulation (i.e., the nutrient value of the feed, animal requirement and feeding systems) should be precise. The integration of the knowledge acquired on meal size, frequency and fibre on the fate of nutrients in the gastrointestinal tract (GIT) in a mechanistic way, as performed for some nutrients such as amino acids, phosphorus and calcium [21,22,23], may be a powerful method to optimise nutrient utilisation.

The present review provides an overview of the factors that influence feedstuff value, the main factors known being dietary fibre and also meal size and frequency. Areas that deserve further investigation to develop precise nutrition systems are identified.

## 2. Natural Feed Intake of the Pig

As characteristics of animal species, meal frequency, size, duration and distribution are reproducible and may vary according to the type of diet offered to the animal [24]. On average, growing pigs eat from 8 to 11 meals per day, with the vast majority during daylight hours [25,26,27]. The number of meals per day can change depending on the physiological status of the pig. For instance, lactating sows eat on average 8.7 meals per day [28], while gestating sows in feeding stations eat on average 1.17 meals per day [29]. Some authors mention that pigs eat continuously without pausing [30]. However, the literature supports that pigs consume several discrete meals per day [31,32,33]. The feeding behaviour of pigs in groups also depends on the social rank of the animal [34].

Dietary factors and non-dietary factors can influence the daily feed intake of the pig. When the dietary concentration of energy is low in a diet, pigs can modulate their intake to meet their requirements [26,35,36]. Moreover, the balance of nutrients in the diet can influence feed intake. Therefore, the protein (amino acid): energy balance should be considered because high-protein levels, high-essential amino acid levels or limiting essential amino acid levels can limit feed intake [37,38]. High levels of branched-chain amino acids (BCAA) in the diet and deficient levels of BCAA can decrease feed intake [39]. Henry et al. (1992) observed that imbalanced Trp: large neutral amino acid levels reduced feed intake through low concentration of serotonin in the hypothalamus [40]. In young pigs, glutamic acid may increase feed intake [41]. Feed processing can also modulate the intake. Reducing particle size is associated with a reduction of feed intake, while liquid feeding stimulates feed intake [37]. Pelleted feed also reduces feed intake compared to mash diets [42]. The presence of mycotoxins in the diet can also reduce feed intake [43]. As for non-dietary factors, gender, body weight, health status, temperature and physical environment can be modulators of feed intake [37]. As the pig grows, feed intake is increased to meet its nutrient requirements for growth. Barrows grow faster than gilts and will thus have a greater feed intake [44]. Activation of the immune system by some health issues causes stress and a decrease in feed intake generated by inflammation [37]. If ambient temperatures increase above the thermoneutral zone, feed intake is reduced [45]. However, pigs can change their feeding behaviour and consume more feed in the morning when it is cooler than in the evening [46]. Feeder space, floor space and group size are also essential to avoid competition between pigs for access to feed. Increasing the number of pigs above the optimum feeder space reduces feed intake [47].

Feed intake is regulated through satiety and satiation signals. Satiety and satiation are two different concepts often taken as the same [32]. Satiation represents the end of a meal and implies all signals related to meal size. Satiety begins after satiation and lasts as long as stimulation for feed intake is absent [32]. When the animal has free access to feed, it eats discrete meals, supporting that satiety inhibits feed intake. Therefore, satiation signals help regulate feed intake more efficiently than hunger signals, which appear when satiety signals are not present anymore [48]. Both satiation and feed intake regulation centres are located in the hypothalamus [42]. The end of a meal is regulated by more than one satiation mechanism such as distension, concentration of digesta or hormonal signals [49]. Therefore, feed intake is controlled by two different mechanisms: (1) satiation signals after a meal and (2) end of satiety signals and the start of hunger signals that stimulate the consumption of a meal.

The number of meals consumed per day by the animal can influence its state of satiety. Indeed, an increase in meal frequency supports satiety by decreasing the variation in the blood glucose concentration and, therefore, low blood glucose, responsible for the initiation of hunger [50]. Moreover, the constant nutrient supply also stimulates satiety through other mechanisms implying gut hormones and osmoreceptors [51].

Prandial correlations measure the strength of the effects of between-meal interval length on the size of subsequent meals (pre-prandial) and vice versa (postprandial) and provide insight into the control of feed intake [52]. The different types of meal regulation (satiation and satiety) can be identified by prandial correlations that combine meal size and frequency concepts. The size of the present meal is positively correlated with the time elapsed before the next meal and is thus a postprandial correlation. The time elapsed before the next visit to the feeder and the size of the future meal are also positively correlated, and it is rather a pre-prandial correlation [31,53].

Pigs demonstrating a pre-prandial regulation of feed intake occupy the feeder longer and less frequently while consuming larger meals. On the other hand, pigs with a postprandial regulation of feed intake frequent the feeder more often and eat smaller meals [53]. Regulation of meal size by pre-prandial correlations involves satiation mechanisms located in the upper part of the digestive system [54]. Pre-prandial correlations are thus based on the stomach and duodenum distension mechanoreceptors and the osmotic receptors located in the duodenum [49,55]. On the other hand, postprandial correlations use mechanisms for regulating meal size linked to satiety located in the lower part of the digestive system [54]. In particular, the ileal brake is involved in the case of postprandial correlations [51]. The ileal brake is a signal inhibiting feed intake, and it is stimulated by the presence of nutrients in the ileum [56]. However, Salgado et al. (2021) report moderate correlations indicating an important variation in feeding behaviour that cannot be solely explained by in-between meal intervals or meal size [57].

## 3. Mechanisms Regulating Meal Size and Frequency

Pigs have an intrinsic motivation to feed themselves to fulfil their nutritional goals for either maintenance, growth or reproduction. Feeding motivation comprises two phases: appetitive and consummatory behaviours controlled by different neuroendocrine systems [58]. As the pig starts eating its meal, positive feedback from the taste and texture of the feed will encourage a continuation of eating. However, as more feed is ingested, signals from the GIT will induce satiation and termination of the meal. The animal will then go on to satisfy other needs such as social behaviour, resting or reproduction. Conversely, when satiety signals start to diminish, hunger signals will intensify, and the pigs will feel the need to consume another meal [58].

Satiety and satiation are controlled in the brain and regulated by several signals from the gut to the hypothalamus [59] by receptors located near the stomach and duodenum to detect the presence of newly ingested feed [49]. However, there are also satiety receptors situated towards the end of the intestine that control the amount of feed consumed during a meal and the time elapsed before the next meal [53]. An effective satiation signal should be initiated at the start of the meal and have a limited duration of action to allow the animal to eat another meal later. There are three main types of regulators to complete the meal: distension, osmotic and hormonal, detailed in the following sections. Depending on the type of diet ingested, the relative importance of the different receptors will be modified. For diets rich in carbohydrates, the osmotic receptors will play a more important role, while hormonal receptors will be more stimulated by a diet rich in protein and fat [38]. In the case of a diet high in dietary fibre, the distension receptors will be more stimulated in the first place due to the more bulky nature of the feed [60]. However, with the fermentation of fibre into short-chain fatty acids (SCFA) that stimulate glucagon-like peptide 1 (GLP-1) and peptide tyrosine–tyrosine (PYY) release [60], hormonal receptors will also play a role in high fibre diets.

### 3.1. Distension

The volume of feed in the stomach activates mechanoreceptors and initiate gastric emptying, which is regulated by the volume of the stomach and also by the volume of the intestines [61,62]. When the chyme passes into the small intestine, the distension receptors become activated, and gastric emptying slows down [55,61]. Mechanoreceptors are located along the afferent branches of the vagus nerve in the muscle layers of the stomach and intestine [51]. Stimulation of motility is proportional to distension of the stomach [63]. Greater distension is observed when the diet contains elements that take longer to digest or are indigestible, such as dietary fibres that are swelled because of their physicochemical properties in water and thus increase digesta volume [55,64]. The presence of hypertonic solution in the intestine, a solution whose concentration in nutrients is greater than the cellular content of the enterocytes, also contributes to distension by drawing water in the intestinal lumen [48]. The distension receptors have a slow effect since they take action 30 min or more after the meal [55]. When the receptors detect distension, the rate of feed intake decreases, so the time to complete a meal increases [64]. Other satiation receptors will come into play to finish the meal.

### 3.2. Osmotic Receptors

The hypertonic digesta newly released from the stomach helps stimulate the end of the meal by activating osmotic receptors present in the lining of the duodenum. Indeed, an increase above isotonicity in the duodenum, above the concentration of nutrients in enterocytes, causes a reduction in meal size irrespective of effects caused by the digesta itself or hypertonic solutions [49]. In Houpt et al. (1983) study, hypertonic solutions containing glucose, xylose, mannitol, sorbitol or NaCl were injected directly into the duodenum [49]. Glucose and NaCl solutions are among the most effective in reducing meal size due to their rapid absorption. Xylose solutions had an intermediate effect, while those of mannitol and sorbitol did not affect meal size. If the same solutions were injected into the portal vein, meal size was not affected, indicating that the receptors are located in the duodenum. There are also osmotic receptors located in the ileum that contribute to the ileal brake. Following stimulation by the digesta or hypertonic solutions, the osmotic receptors send a nervous influx to the rhombencephalon through the vagus nerve [51,55]. The influx is sent to the hypothalamus, responsible for sending nervous signals of gastric inhibition to stop gastric emptying [55].

### 3.3. Hormonal Receptors

Several peptides can be secreted by enteroendocrine cells and act on different receptors located on the afferent branches of the vagus nerve (Table 1). The effects will be different depending on the hormone secreted [51]. Most hormones are anorexigenic, i.e., responsible for ending the meal by reducing feed intake except for ghrelin that is orexigenic [38].

Unlike all other peptide hormones, ghrelin helps increase meal size. Its action is particularly important in diets diluted by components reducing energy density, such as dietary fibre [51]. With these diets, the secretion of ghrelin causes the animal to consume a larger volume to obtain a sufficient level of energy. In the stomach and duodenum, ghrelin is secreted by enteroendocrine X and A-like cells [51]. The intensity and duration of the response depend on the calories ingested by the animal. Mechanical distension of the stomach does not stimulate ghrelin secretion because the blood glucose level plays a key role in ghrelin release [59]. The ghrelin effect can also be suppressed by feedback from the hindgut via excessive fermentation [38]. Ghrelin regulates energy homeostasis and stimulates the release of growth hormone [59,65]. Along with stimulating feed intake, ghrelin can increase blood glucose, gastric movement, gastric acid secretion and turnover of the gastric and intestinal mucosa [59]. The functions of ghrelin are antagonists to those of leptin [59,65]. Soluble non-starch polysaccharides (S-NSP), such as guar gum, can increase plasma ghrelin levels [66].

Cholecystokinin (CCK) is produced by enteroendocrine I cells located in the stomach and duodenum. The CCK is important for meal size regulation, gallbladder contractions, gastric emptying, intestinal motility, and gastric and intestinal secretions [51,67]. Responses to CCK depends on the dose, and to be efficient, CCK should be released at the onset of the meal [51,55]. The CCK limits meal size, acting as a satiety signal [67,68]. Dietary fibre and, more specifically, β-glucan can enhance the duration of the anorexigenic response of CCK [38].

Glucose-dependent insulinotropic polypeptide (GIP) is secreted by intestinal K-cells present in the duodenum and proximal jejunum in response to glucose and lipid absorption. GIP is partly responsible for postprandial insulin release [38]. The GIP can regulate feed intake by regulating nutrient intakes such as glucose, amino acids and fatty acids [69]. Other effects include stimulating fat storage and optimising nutrient delivery to tissues [69]. Postprandial net portal appearance (NPA) of GIP is lowered by 81% when slowly digestible starch is added to the diet of pigs [70].

GLP-1 is released from L-cells located at the distal part of the intestine. Its secretion is meal dependent, and thus plasma levels are lower between meals [71]. GLP-1’s primary effect, like GIP’s, is to stimulate insulin secretion [71]. Other effects include reduction of intestinal motility and secretions in addition to decreasing gastric emptying. Combined, these effects are responsible for the ileal brake, an inhibiting signal by reducing feed intake [51,69]. In contrast to the other incretins that exert their action in the upper part of the gut, GLP-1 plays its role later on in the digestion process in the ileum. Dietary fibre can increase the number and differentiation of L cells in the jejunum, ileum and colon and then increase GLP-1 secretion [72,73,74]. Consequently, feed intake is decreased because of the greater levels of GLP-1 caused by dietary fibre [60]. Postprandial net portal appearance (NPA) of GLP-1 is lowered by 36% when slowly digestible starch is added to the diet of pigs [70]. Fermentable non-starch polysaccharides (fNSP) have been shown to increase the secretion of GLP-1 in the intestinal mucosa and also in the blood [75].

PYY is secreted from L-cells located in the ileum, colon and rectum and is signalling energy homeostasis. Its release follows a cycle with an increase in anticipation of a meal and then a further increase with the onset of meal consumption [38]. Its secretion is then directly related to the energy content of a meal, and plasma PYY is reduced in between meals [76]. The energy density of the feed strongly influences the release of PYY making dietary fibre, resistant starch and lipids the principal nutrients increasing its secretion [51,77]. The effect of dietary fibre and resistant starch is, however, indirect. Dietary fibre increases fermentation and thus SCFA production. Those SCFA then bind to L-cells, which stimulates PYY secretion [60]. The PYY is implied in the ileal brake and plays a role in gut function and lipid absorption [71,78]. Additionally, PYY can delay small intestine mean retention time and gastric emptying [79]. Fermentation of dietary fibre into SCFA can stimulate the release of PYY and thus influence feed intake in the long term [80].

Apolipoprotein A-IV (apo A-IV) is responsible for fatty acids transport but can also reduce meal size. Combined with enterostatin, these two peptides can decrease selective fat intake [51]. Apo A-IV is paired with chylomicron and secreted in the small intestine, mainly in the jejunum [78]. By modifying the gastric and intestinal functions, Apo A-IV inhibits intestinal motility [78]. Apo A-IV is stimulated by lipid intake and can limit feed intake [78].

Leptin plays an important role in the central regulation of meal size and is mainly secreted by adipocytes [78,81]. Leptin manages energy balance in the hypothalamus and other regions of the brain [51,82]. Leptin also allows adaptation to fasting when carbohydrate metabolism switches into fat metabolism and insulin levels are low [82]. Low leptin plasma levels signal the brain that energy supplies are low, while high levels indicate enough energy is available [81]. High leptin levels can then reduce feed intake [82]. A leptin injection at the intracerebroventricular level reduced meal size and increased the secretion of growth hormone [42,83]. Many other factors are secreted by adipocytes, such as adipokines, that can influence feed intake [84,85]

## 4. Impact of Meal Size and Frequency on Transit Time and Digestibility of Nutrients

The impact of meal size on digestibility is mainly due to its effect on gastrointestinal transit (Table 2). A large meal causes faster gastric emptying during the first 30 min [61]. Instead, the rapid arrival of the hypertonic digesta in the duodenum leads to its distension. Mechanoreceptors and osmotic receptors then send signals to stop gastric emptying [61]. Furthermore, intestine distension caused by a large meal causes contractions that move the meal forwards down the intestinal tract [86]. The retention time is thus reduced, thereby potentially decreasing digestibility [87]. Indeed, when the pig was fed a quantity of feed intended to meet 1× its maintenance energy requirements in two meals per day, total tract transit time is 52.5 h instead of 35.3 h when the pig receives a quantity of feed covering 2.5× its maintenance energy requirement in two meals per day [87]. When transit time is too short, digesta does not have enough contact time with digestive enzymes, nutrient absorption zones and the microbiota for fermentation, decreasing ileal and total digestibility [88].

An increase in meal frequency can increase nutrient digestibility by the more continuous flow of digesta in the digestive tract, which increases the production of certain endogenous enzymes [89]. Hee et al. (1988) observed that secretions of amylase, trypsin and chymotrypsin were increased with feeding twice or three times daily [10].

Mroz et al. (1994) fed five cannulated pigs their daily feed allowance in either one, two or seven meals per day [90]. Apparent total tract digestibility of calcium, tryptophan and isoleucine were lower for pigs fed only once a day than pigs fed several times a day. In the same study, apparent ileal digestibility was increased for phytic acid, cystine, arginine, isoleucine and phenylalanine when meal frequency increased from one to two meals per day. However, apparent ileal and total tract digestibility of nutrients did not differ between pigs fed twice or seven times per day. Jia et al. (2021) did not observe differences in the apparent total tract digestibility (ATTD) of dry matter (DM), ash, crude fibre (CF), ether extract (EE), nitrogen-free extract (NFE), total carbohydrates (CHO) and organic matter (OM) when pigs were fed once, three times or five times per day [91]. However, ATTD of crude protein (CP) was increased when pigs were fed three or five times per day instead of once per day [91]. In the same study, pepsin secretions were increased with more meals per day, indicating that the GIT of pigs adapts to the feeding conditions to increase digestive capacity [91].

In general, the motility of the intestine in the duodenum is increased following the ingestion of a meal. On the other hand, the duration of the myoelectric complex responsible for intestinal motility varies according to the frequency of meals. In pigs receiving only one meal per day, the pattern of postprandial intestinal contractions lasted for 6 h, with an average of 13 myoelectric complexes per day [92]. However, the number of myoelectric complexes is increased to 16 in pigs fed twice a day [92]. In addition, with two meals a day, the pattern of postprandial intestinal contractions is spread over 2–3 h. When comparing pigs fed once or twice a day with pigs with ad libitum access to feed, the motility of the stomach and intestines is reduced in pigs receiving a limited number of meals [92]. However, a decrease in motility does not affect the ileal digestibility of crude protein and amino acids [93].

In pigs with ad libitum access to feed, the microorganisms in the colon receive a greater quantity of undigested residue, which can reduce their fermentation efficiency and thus decrease the total digestibility of dry matter and energy [93]. However, fermentation efficiency also depends on the structural composition of the dietary fibre. Xu et al. (2020) also found similar results in pigs with ad libitum access to feed with a high dietary fibre content containing whole wheat grain and wheat bran [94]. In this study, arabinoxylan (AX) was degraded through the GIT up until the mid-colon. Butyrate production was increased modestly by the high consumption of fibre in the diet [94]. A study by Glitsø et al. (1999) looked at the degradation of AX in different rye diets with pigs being fed a restricted amount of feed twice a day [95]. They found that AX of rye endosperm was degraded between the proximal ileum to the caecum, whereas AX from rye aleurone was degraded from the proximal ileum to mid colon. Whole rye was degraded from the caecum to the mid colon [95]. Therefore, structural characteristics of AX influence their degradation in the intestine more than transit time itself. The AX was degraded similarly in Xu et al. (2020) and Glitsø et al. (1999) for two different feed intake levels [94,95]. The degradation level is interesting because it can affect the substrate supply to the microbiota and thus the fermentation capacity. Notably, the quantity of substrate available depends on pre-ileal digestibility. Moreover, the GIT can adapt to high dietary fibre intake and become more efficient in fermenting them [96].

## 5. Impact of Meal Frequency on Blood Profile, Metabolism and Body Composition

Among the impacts of meal frequency on energy metabolism, we first find the thermic effect of food, which describes the energy required for digestion and subsequent deposition of excess nutrients occurring between 0 and 8 h after the ingestion of a meal. With increasing meal frequency, the thermic effect of food may decrease mainly due to a decrease in insulin fluctuation [97]. Indeed, fluctuations in blood glucose and insulin concentrations are attenuated with increased meal frequency [50].

In addition, meal frequency modulates metabolism, particularly the flux of metabolites in and out of storage. With two meals a day, the intermediate metabolism must alternate between deposition and release of nutrients, increasing lipogenesis in pigs adipose tissue [98]. Reduced body fat is observed with multiple meals per day combined with induction of cataplerosis (use of citric cycle intermediates for amino acid synthesis) during the pre-prandial period, which leads to increased protein synthesis [99]. Blood glucose and amino acid levels stabilise with several meals a day with a decrease in the postprandial insulin concentration [89,98]. Stabilized blood glucose and amino acids levels are due to decreased stomach distension with smaller, more frequently served meals that reduce the fluctuations of gastric emptying. As a result, starch and protein are transported at a more constant rate to the intestine for digestion into glucose and amino acids [100].

Over an extended period, a decrease in energy loss with increased meal frequency can increase fat deposition in adipose tissue [97]. This claim is supported by a study in humans indicating that less fat from breakfast is oxidised with the consumption of three meals a day rather than two. Provided the same energy intake, more fat is thus stored when humans ingest three meals a day [50]. Since the interval between meals is greater with the consumption of two meals per day, more fat from breakfast is metabolised during the day rather than deposited. Decreased body fat in human subjects consuming one meal a day was accompanied by increased LDL and HDL cholesterol levels [101].

In summary, increased meal frequency per day increases lipogenesis [50]. However, conflicting results are found in the literature. LeBlanc and Diamond (1986) indicate that a reduction in the number of meals leads to increased lipogenesis [102]. In their study with dogs, they observed a greater energy expenditure for digestion with increased meal frequency. Additionally, they noted that a major part of energy expenditure in the form of heat comes from the effect of the palatability of the food. In dogs, high palatable foods stimulate the animal and cause hyperventilation, which causes a loss in energy. In human subjects fed a low meal frequency (three meals per day without snacks), lipogenesis increased, absorption of fat and glucose in the intestine was faster, and glycogen synthesis was increased [103]. Fat storage is more important than protein deposition in rats fed few meals a day [104], indicating that by increasing the number of meals per day, energy and amino acid supplies are better synchronised, which supports an optimised protein deposition and leaves less energy available for fat deposition [99,105]. With two meals per day, more nutrients in circulation cannot be deposited efficiently in muscle tissue leaving more nutrients available for adipose tissue or liver for fat synthesis.

## 6. Impact of Fibres on Digestive Function and Metabolism

Several definitions of fibre have been created by numerous authors, but the Codex Alimentarius Commission proposed a consensus definition of dietary fibre as carbohydrate polymers with ten or more monomeric units, which are not hydrolysed by the endogenous enzymes in the small intestine of humans [106]. However, this definition is vague, does not describe the properties of fibres and is not adapted to animal science. Dietary fibre sources in animal science can be then classified by chemical composition and structural composition. Moreover, dietary fibres are commonly classified by polymers and oligomers depending on their physicochemical properties, solubility, viscosity and fermentability [107]. Because of its non-digestibility in the small intestine and resistance to fermentation by the microbiota and its physiochemical properties, dietary fibre can modify transit time in different parts of the GIT [16]. Thus, dietary ingredient composition is a concern nowadays because feeds can include many fibre-rich ingredients to replace part of the corn and soybean meal that are commonly used in Eastern Canada and the United States. Additionally, we know little about the possible interactions between dietary fibre content and the frequency and size of meals on the digestive capacity of pigs.

Most dietary fibre is fermented by microbiota that produces SCFA. Generally, soluble fibre is more easily and rapidly fermented than insoluble fibre [76]. Therefore, increased absorption of SCFA can prolong satiety through prolonged postprandial energy supply. Moreover, SCFA can link to free fatty acid receptors (FFA2 and FFA3) present on L-cells, inducing PYY and GLP-1 release [60,76].

Especially viscous dietary fibre can stabilise blood glucose with a slower gastric emptying rate leading to slower glucose absorption [108]. de Leeuw et al. (2005) fed cows with a diet containing either a low (173 g/kg) or high (378 g/kg) content of fNSP and infused fNSP in the caecum or glucose in the blood [109]. Infusions of fNSP and also of glucose prevented a drop of blood glucose in between meals, thereby prolonged satiety.

## 7. Fibre Content in Pig Diets

**Figure 1:**
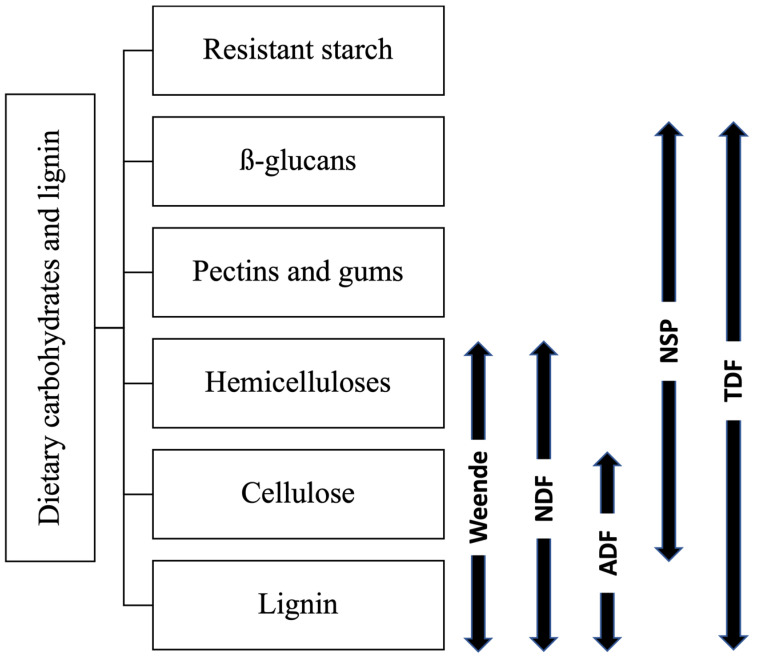
Dietary fibres determination methods and the fractions they include (adapted from NRC 2012) Errows represent the dietary carbohydrates that are included in the analysis (Weende analysis only includes part of the cellulose).

Various types of analyses can measure fibre content in pig diets (Figure 1). One of the oldest methods of analysing fibre content is the Weende global analysis. Carbohydrates are divided between non-nitrogenous extract and crude fibre, according to Weende global analysis [110]. The crude fibre method uses acidic and then alkaline solutions to remove protein, sugars and fat from the sample. However, the crude fibre method is now set aside because it does not allow accurate determination of fibre content. Only cellulose, lignin and part of hemicellulose are determined in the Weende global analysis [111].

The detergent methods were developed to better characterise diets for ruminants. These methods are also widely used in monogastric nutrition [111]. In the case of neutral detergent fibre (NDF), sodium sulfite and anionic detergent (EDTA) are used to remove the proteins. Starch is removed by the addition of α-amylase and fat by cleaning with acetone. At the end of the assay, the insoluble fibre remains, namely cellulose, hemicellulose and lignin. The soluble fibres have been solubilised by the detergent and are no longer present in the final sample [111]. For acid detergent fibre (ADF), a solution of sulfuric acid and anionic detergent is used to remove protein and starch from the sample. The fat is removed beforehand with an acetone wash. In this case, only cellulose and lignin are measured. However, these two methods cannot be used to determine soluble fibres content because these are dissolved in hot detergents and thus removed from the sample [111]. However, the determination of soluble fibres would allow a better understanding of the dynamics within the digestive system, particularly in the stomach and small intestine, given that their solubility and their quantity in the diet affects the volume and rate of digesta passage in the digestive tract [112].

A method that allows a more detailed analysis of fibre is the non-starch polysaccharides (NSP) determination. NSP include β-glucans, pectins, gums, hemicelluloses and cellulose. The most widely used method to determine the NSP is the Englyst method, an enzymatic–gravimetric analysis that measures NSP fibre [113]. The Uppsala method measures NSP but also includes measurements of lignin. Lignin is important in animal nutrition as all feedstuffs, and co-products in particular, are rich in lignin. The Uppsala method can thus measure total dietary fibre (TDF) by combining NSP and lignin determination [111]. A combined Englyst and Uppsala procedure was applied by Bach Knudsen (1997) to analyse a wide variety of feedstuffs for carbohydrates, including soluble and insoluble non-cellulosic polysaccharides (hemicellulose), cellulose and lignin [114]. The analysis consists of three parallel procedures (A, B and C). In procedure A, the sample is first digested with α-amylase and amyloglucosidase in vitro. The digested sample is then mixed with ethanol to precipitate soluble fibres. The mixture is then centrifuged, washed and dried to obtain a starch-free residue at the bottom of the tube. Sulfuric acid (H_2_SO_4_ 12 M) is then added to the residue to swell cellulose. Then, all NSP are hydrolysed to monosaccharides with 2M H_2_SO_4_ for one hour in a boiling bath. The aldehyde group of neutral sugars is reduced to obtain sugar alcohols, which undergo derivatisation to acquire alditol acetate. The determination of alditol acetate is done by gas chromatography. Uronic acids are determined separately by colourimetry and Klason lignin as the residue following acid hydrolysis. In procedure B, the swelling of cellulose with 12 M H_2_SO_4_ is left out to hydrolyse the non-cellulosic polysaccharides (NCP) directly with 2 M H_2_SO_4_. As for procedure C, the soluble fraction (S-NSP) is extracted using a phosphate buffer at 100 ˚C at neutral pH before ethanol precipitation and acid hydrolysis to only keep the insoluble fraction (I-NSP) [77]. The different fractions can then be calculated as follows:

Cellulose = NSPGlucose (12 M sulfuric acid) − NSPGlucose (2 M sulfuric acid)(1)

NCP = rhamnose + arabinose + xylose + galactose + glucose + uronic acids(2)

S-NSP = Total NCP − I-NCP(3)

TDF = NSP + lignin(4)

The NSP method is more precise for measuring the content of soluble fibres. However, it does not include oligosaccharides that do not precipitate in ethanol and are eliminated during cleaning [111]. It is possible to determine the oligosaccharides by doing the direct method, which consists of not treating the sample with α-amylase and amyloglucosidase and not adding ethanol and starting at the sulfuric acid treatment directly. In this manner, it is possible to determine the oligosaccharide portion by subtracting the glucose and total NSP obtain by the original method from those obtained by the direct method.

The majority of NSP are contained in the cell walls of plants such as cellulose, β-glucan, arabinoxylans, rhamnogalacturans, galactans and pectins. The others, such as fructans and inulin, are not found in the cell wall but rather inside the cell. The cell wall of wheat grain can contain up to 60–70% of NSP [107]. These polysaccharides are not digestible because pigs do not have the endogenous enzymes associated with this type of substrate [115]. However, some fibres can be fermented by bacteria either in the small or large intestine. Generally, large and branched polymers present in lignified tissues are less fermentable than smaller polymers or oligomers [107]. The resulting products are lactic acids and short-chain fatty acids that can then be used for energy metabolism.

## 8. Impact of Fibres on Feed Digestion

Although fibre can be partially broken down by the pig’s gut microbiota, the majority of fibre remains undigested, in addition to acting negatively on the digestion process.

### 8.1. Dilution of Energy Content

Fibre, as it cannot be digested by pigs, reduces the total tract digestibility of energy, thereby reducing metabolisable energy by decreasing the energy density of the feed [112,116,117]. The digestible energy (DE) content is reduced by 1% for each 10 g increase of NDF fibres per kg of feed [118]. Other authors also support this observation and have noticed a 32% decrease in apparent ileal digestibility of energy when dietary NSP content increased from 83 to 193 g/kg [119]. In addition, the ingestion of fibres has a bulking effect in the stomach that limits the feed intake of pigs [119]. Bulking effect is caused by the insoluble fibres that can retain a large amount of water, but also by soluble fibres that create a viscous gel [107]. Increasing dietary water-holding capacity from 1.56 to 4.37 g water/g DM was associated with a 10% increase in mean retention time [66].

### 8.2. Viscosity

**Figure 2:**
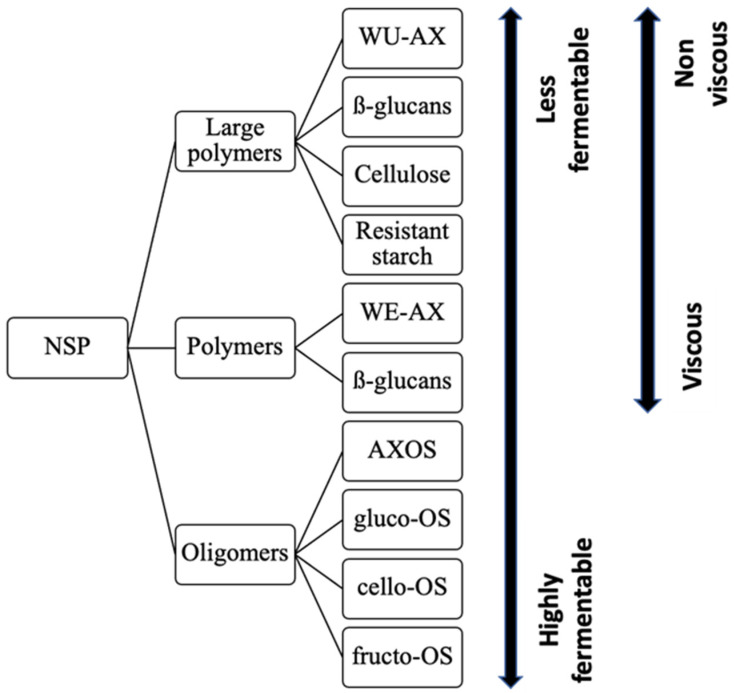
NSP and their physiochemical properties (adapted from Bautil and Courtin, 2019 [107]). Errows represent a scale of viscosity and fermentability. AXOS: arabinoxylooligosaccharides, OS: oligosaccharides, WE-AX: water-extractable arabinoxylan, WU-AX: water unextractable arabinoxylan.

Soluble fibre leads to the formation of a gel that traps water and increases digesta viscosity (Figure 2). The larger volume with retained water causes an increase in abrasion with the intestinal wall and thus a greater loss of epithelial cells [119]. In wheat and rye, viscosity is mainly caused by water-extractable AX (WE-AX) depending on its degree of substitution, the pattern of the xylan backbone and chain length [107]. However, in barley and oats, mixed-link β-glucans are the most important contributor to viscosity [120]. Viscosity is a function of the polymer in solution and the molecular weight of the molecule [120]. The molecular weight of mixed-link β-glucans is greater than AX, making it more viscous in solution [120]. The interaction between the fibres and the intestinal wall increases mucin production and thus the endogenous losses of several nutrients contained in mucin, such as amino acids [121,122]. Moreover, an elevated content in NSP can stimulate secretions of endogenous enzymes, electrolytes and bile to compensate for the lack of absorption and nutrient diffusion caused by the viscosity [107,120]. The interaction between soluble fibre and intestinal mucus reduces the epithelium permeability, preventing an efficient absorption of nutrients [122]. The formation of gels also reduces digesta contact with bile preventing adequate micelle formation for the action of lipases [74]. Some authors support that a more viscous digesta reduces the contractions of the intestine, thereby slowing down transit and reducing the supply of substrate to bacteria by reducing the mixing of intestinal content [123]. On the other hand, other authors mention that the increase in volume in the digestive system due to viscosity causes a faster digesta transit decreasing the absorption of glucose, triglycerides and cholesterol [74,124]. These discrepancies in the literature are probably due to the fact that it is challenging to measure digestive transit and the lack of diets tested for functional characteristics and variability due to fibre content and functionality and meal size and frequency.

## 9. Impact of Exogenous Enzymes on Nutrient Utilisation

Exogenous enzymes such as carbohydrases like xylanase, β-glucanases or phytases can be added to the diet to release nutrients embedded in the fibre matrix or phytate. New generation phytase is a well-known and widely-used enzyme in pig diets that are becoming more and more effective due to better adaptation to digestive tract conditions [125,126]. However, while the effects of xylanase are well documented in broilers [127,128], its effects in pigs are more variable. This difference can be explained by transit time that is slower in pigs than in broilers [129]. Part of the variation can also come from the diet and its ingredient composition. Several authors have noted an increased digestibility with xylanase addition in wheat-based diets [130,131] that are more typical for pig diets than broiler diets. However, other authors have found that xylanase had little to no effect on the digestibility of diets containing DDGS [132]. Xylanase may reduce digesta viscosity in pigs fed wheat or rye-based diet [133]. Conditions of pH or retention time can vary when changing meal size and frequency [15,86], which then affects the efficiency of exogenous enzymes. Both xylanase and phytase have optimal activity conditions, which means they need to degrade their substrate in a relatively short period from when the feed is first moistened in the mouth to the distal ileum [129].

### 9.1. pH

Meal frequency can modify the pH in the digestive tract. The presence of feed in the stomach stimulates the secretion of gastrin and histamine, which in turn cause the release of hydrochloric acid [86]. However, the feed can also act as a buffer in the stomach. Pig diets are commonly containing limestone to provide calcium and monocalcium phosphate to provide phosphorus. Both of these ingredients exert an important buffering capacity at pH 3 [134]. Feed form can also affect the stomach pH. Indeed, pH is lower when pigs are fed pelleted or finely ground diets than coarse mash diets [135]. A higher number of meals per day may stabilise gastric pH at a higher level. The average pH in the stomach is around 4 and increases slightly with meals and decreases when the stomach becomes empty. The pH in the small intestines is about 6–7.5. Xylanase is sensitive to pH conditions below 2.5. In addition, its window of activity is between pH conditions varying from 4 to 6 [129]. However, these pH optimums can differ depending on the type of xylanase used [136]. More generally, xylanase efficiency depends on the pK_a_ of its catalytic residues. As the pH changes, the enzyme can fold or unfold, which change exposure to the catalytic site and then efficiency [136,137]. Each xylanase has thus a different pH optimum, and to our knowledge, there is limited literature listing various types of xylanases and their optimal pH conditions. Conversely, phytase is inactive at the pH conditions of the intestine following the duodenum, its window of activity for in vivo conditions being between 2.5 and 4.5 pH [20,129]. Thus, a high pH in the stomach would allow a better degradation of fibres but would decrease phytase activity and reduce the digestibility of protein [129].

### 9.2. Retention Time

With increased retention time, exogenous enzymes have more time to hydrolyse their substrate. On the other hand, the longer enzymes remain in the tract, the longer it is exposed to a hostile environment. Exogenous enzymes are then exposed longer to endogenous proteases secreted by pigs, such as pepsin, trypsin and chymotrypsin [138]. Since exogenous enzymes are proteins, prolonged exposure to proteases decreases their activity. Phytase derived from *E. coli* were more resistant to degradation by endogenous protease [20].

When a limited number of meals is served to pigs, more feed is stored in the stomach because gastric emptying is slower [129]. Van Leeuwen and Jansman (2007) observed that the retention time in the stomach varies between 3 and 4 h when pigs are fed twice a day [15]. In contrast, Wilfart et al. (2007) noted that the retention time in the stomach is of an hour when animals are fed every four hours [139]. Since phytase is more efficient under pH conditions of the stomach, prolonged retention time at the stomach level could be advantageous and provide more degradation of phytic phosphorus. However, the pH of the stomach needs to be optimal for phytase degradation; otherwise, a longer retention time in the stomach won’t improve phytate degradation.

Increasing dietary insoluble fibre reduces retention time [140]. The faster transit gives exogenous enzymes less time to exert their action. Decreased efficiency of enzymes is then observed [138]. With a dietary NDF content of 15% or less, retention time decreases, but a further augmentation of fibre content in the diet causes augmentation in retention time because of stomach distension which inhibits gastric emptying [141].

## 10. Conclusions

In conclusion, meal size and frequency can affect ileal and total tract digestibility of nutrients through modulation of GIT condition (pH and transit time), metabolites (glucose and SCFA) and hormones (GLP-1 and PYY). Depending on the dietary composition, the dietary fibre will influence distension receptors in the stomach and thereby control the feed intake. Viscosity and water-binding capacity properties of fibres also affect transit time, digestibility of nutrients and thereby the feed intake. A change in digestive tract conditions induced by changing meal size or frequency can modify pH and transit time and therefore influence the efficiency of exogenous enzymes. As shown in this review, the effects of diet type and fibre and meal size and frequency on transit time can be variable among studies. Measurement of transit time should be assessed more frequently along with digestibility in trials to fill in the gaps in the literature on the effect of meal size and frequency and fibres. With a better understanding of the underlying mechanisms involved in digestion, the development of more precise feeding systems will be possible in the near future.

## Figures and Tables

**Table 1:** Feed intake regulating hormones secretion site and their effects.

Hormone	Secretion Site	Location	Effects
Ghrelin	X- and A-like cells	Stomach and duodenum	Increases meal size Energy homeostasis Release of growth hormone Increases gastric movement
CCK	I cells	Stomach and duodenum	Decreasing meal size Stimulates gallbladder contractions Reduces gastric emptying and intestinal motility
GIP	K cells	Duodenum	Stimulates insulin release and fat storage Optimises nutrient delivery to tissues
GLP-1	L cells	Ileum and colon	Decreases feed intake Stimulates insulin secretion Reduction of intestinal motility Decreases gastric emptying
PYY	L cells	Ileum and colon	Regulates energy homeostasis Decreases gastric emptying and intestinal motility
Apo A-IV	In the jejunum	Jejunum	Inhibits intestinal motility Reduces feed intake
Leptin	Adipocytes	Adipose tissue	Regulates energy balance High levels reduce feed intake

Apo A-IV: apolipoprotein A-IV, CCK: cholecystokinin, GIP: glucose-dependent insulinotropic polypeptide, GLP-1: glucagon-like peptide 1, PYY: peptide tyrosine tyrosine.

**Table 2:** Effect of meal size and frequency on mean retention time (MRT), digestibility, fermentation and metabolism.

	Meal Type	Effect on MRT	Effect on Digestibility and Fermentation	Effect on Metabolism	References
Meal size	Large meal	Reduced MRT	Decreased digestibility		Roth and Kirchgessner, 1985 [87]
	*Ad libitum*		Reduced fermentation		Chastanet et al., 2007 [93]
			Fibre degraded from distal ileum to mid-colon Depends on AX structure		Xu et al., 2020 [94] Glitsø et al., 1999 [95]
Meal frequency	Increased number of meals per day	Continuous flow of digesta		Secretions: Amylase ↑ Trypsin ↑ Chymotrypsin ↑	Hee et al., 1988 [10]
			ATTD of Ca, Trp, Ile, Cys, Arg, Phe and phytic acid ↑		Mroz et al., 1994 [90]
			ATTD of CP ↑	Pepsin secretions ↑	Jia et al., 2021 [91]
		Reduced fluctuation of gastric emptying	Constant rate of glucose and amino acids digestion	Blood glucose ↓ Insulin fluctuation ↓	Smeets et al., 2008 [50] Palmer et al., 2009 [100]
				Lipogenesis ↑	Smeets et al., 2008 [50] Tai et al., 1991 [97]
	Limited number of meals per day	Reduced motility of intestines and stomach			Ruckenbusch et al., 1976 [92]
				Lipogenesis ↑	LeBlanc et al., 1986 [102]

↑: Increase, ↓: Decrease.

## Data Availability

Not applicable.
